# An Uncommon Presentation of a Rare Syndrome: Parsonage-Turner Syndrome Presenting With C8 Root Involvement

**DOI:** 10.7759/cureus.28065

**Published:** 2022-08-16

**Authors:** Mustafa H Temel, Elem İnal, Nilgün Mesci, Duygu Geler Külcü

**Affiliations:** 1 Physical Medicine and Rehabilitation, Üsküdar State Hospital, İstanbul, TUR; 2 Physical Medicine and Rehabilitation, University of Health Sciences, Haydarpasa Training and Research Hospital, İstanbul, TUR

**Keywords:** rehabilitation, pain, c8 nerve root, neuralgic amyotrophy, parsonage-turner syndrome

## Abstract

Parsonage-Turner syndrome or neuralgic amyotrophy is a rare syndrome that presents with sudden and severe pain in the shoulder girdle and may cause loss of muscle strength, atrophy, and sensory deficits. We discuss the clinical and electroneuromyographic findings in a young woman who presented with left-sided arm pain and was diagnosed with Parsonage-Turner syndrome with C8 root involvement, which is a rare presentation of the disease.

## Introduction

Parsonage-Turner syndrome, also known as neuralgic amyotrophy, was first described by Parsonage and Turner in 1948 [1]. The disease is a syndrome that initially presents with a sudden onset of pain in the shoulder girdle and can cause paresis and atrophy within days or weeks, with slow and sometimes incomplete recovery [2,3]. Typically, the pain does not change with a change in positioning and can disrupt sleep. The pain is usually self-limiting and lasts one to two weeks except in rare cases [4]. Males are more commonly affected than females, and the prevalence rate of the disease is 1.64 per 100,000 people [5]. The most common sites of involvement are the upper trunk, suprascapular nerve, long thoracic nerve, and axillary nerve, while the least commonly involved peripheral nerves are the ulnar and radial nerves [4]. In this report, we discuss a female patient who was admitted to our clinic with complaints of left-sided arm pain, weakness of the left hand, and numbness on the inner side of the arm affecting the fourth and fifth fingers; she was eventually diagnosed with Parsonage-Turner syndrome after clinical examination and electroneuromyographic studies.

## Case presentation

A 26-year-old woman was admitted to our hospital with complaints of arm pain on the left side, loss of strength in the left hand, and numbness in the fourth and fifth fingers. The patient stated that her complaints had been going on for about six months and that she had been exposed to intense emotional stress beforehand. Three days after the onset of pain, she had started to lose strength in her left hand, especially in the fourth and fifth fingers, and become unable to do her daily work. She had been prescribed medications for pain palliation at various clinics, but her pain had not eased. The differential diagnoses in this case included cubital tunnel syndrome, cervical radiculopathy, entrapment neuropathy, paraneoplastic syndrome, thoracic outlet syndrome, and Parsonage-Turner syndrome.

**Figure 1:**
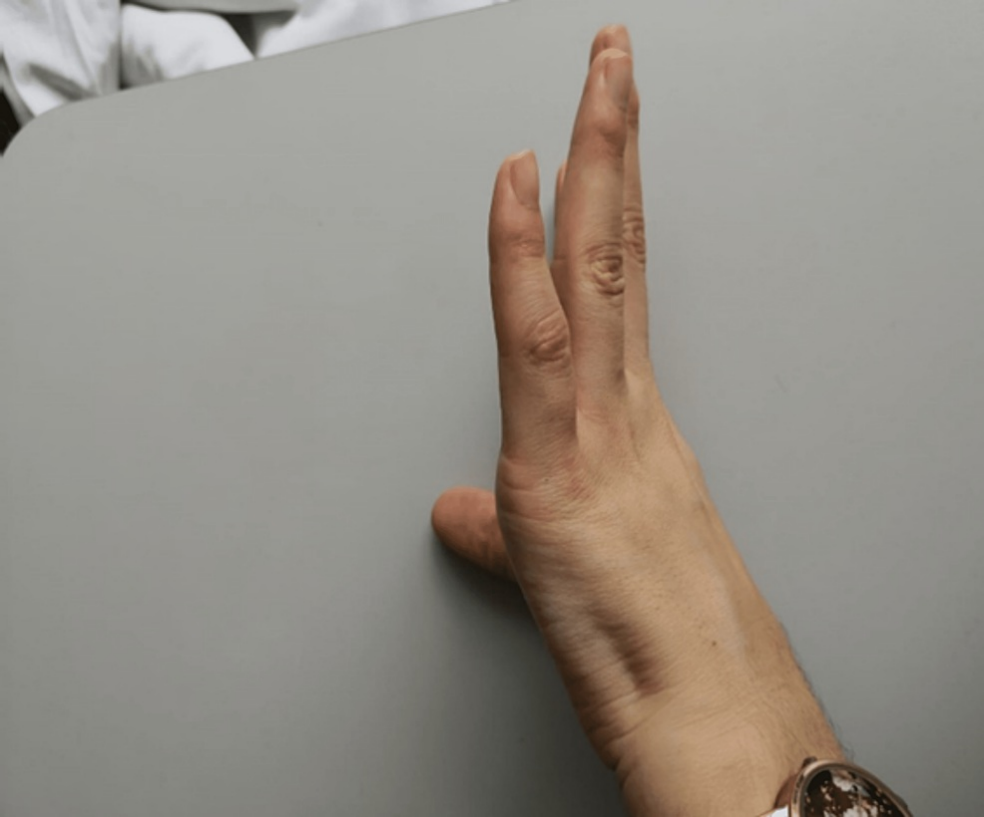
Atrophy in the abductor digiti minimi muscle

The patient's personal and family history was unremarkable, and her general condition was good. She was conscious, oriented, and cooperative. Her cardiovascular system examination, abdominal examination, and pulmonary examination were normal. The neck examination of the patient showed that the range of motion of the joints was full and painless; compression and distraction tests were negative. The shoulder examination revealed that joint movements were full and pain-free, and muscle strength was 5/5. The elbow and wrist examinations demonstrated joint ranges of motion to be full and muscle strength to be 5/5. Adson's maneuver was negative. The hand examination showed muscle weakness in the intrinsic left-hand muscles, hypoesthesia in the left-hand hypothenar region, and atrophy in the abductor digiti minimi muscle (Figure 1). Deep tendon reflexes were normal, and no pathological reflex was detected.

The patient's cervical MRI showed minimal findings at the C5-6 and C6-7 levels. The first electromyography (EMG) ordered showed low ulnar nerve compound muscle action potential (CMAP) amplitude, but sensory conduction was normal, and so was needle EMG examination. No electromyographic findings consistent with the discopathies observed in the cervical MRI were observed, and a repeat EMG after three weeks was recommended for a definitive diagnosis. Cranial MRI, shoulder MRI, and elbow and wrist MRI were reported as normal, and no abnormal findings were documented in laboratory studies and serologies (such as complete blood count, liver and kidney panels, autoimmune and rheumatologic markers, B12, folic acid, and viral tests). Radiological imaging methods excluded a possible pulmonary pathology, and the diameter of the subclavian artery was reported as normal on the Doppler ultrasound examination performed during Adson's maneuver. Axillary ultrasonography, requested for a possible compressive mass or fibrous band, did not reveal any pathological findings. In the control EMG of the patient, the compound muscle action potentials of the left ulnar and left radial nerves were found to be of slightly low amplitude, and motor conduction studies of other nerves examined in both upper extremities were normal. Needle EMG showed prolonged polyphasic motor unit and denervation potentials in the left C8 root innervated muscles and normal ones showing advanced dilution. Other muscles examined were normal. The findings were consistent with C8 radiculopathy with axon damage on the left, and the correlation with the clinical findings was consistent with the diagnosis of Parsonage-Turner Syndrome. After the diagnosis, transcutaneous electrical nerve stimulation (TENS) therapy and oral pregabalin treatment were initiated for pain control. Range of motion and strengthening exercises were performed for upper extremity muscles. Electrical stimulation (ES) was applied to atrophied muscles. At the end of three weeks of treatment, the patient's pain improved and muscle strengthening was observed, but atrophy persisted. The patient was educated about home exercises and discharged with instructions for a follow-up visit.

## Discussion

Although the pathophysiology of Parsonage-Turner syndrome has not been fully explained, various risk factors and a predisposing condition can be identified in 28-83% of patients [4]. The most important risk factor for the development of the syndrome has been described as a viral disease, followed by immunization [6]. A rare hereditary form has also been reported. The aims of the rehabilitation process of the disease involve providing favorable conditions for nerve healing, preventing the development of contracture, and pain management [3]. While most patients have 80-90% of their previous muscle strength restored within two to three years, approximately 70% may have a residual motor and sensory deficit. This makes patients more prone to a possible musculoskeletal injury [7]. The rate of recurrence of the disease has been reported to be 5% [8].

Amyotrophic lateral sclerosis, primary or metastatic brachial plexus tumors, entrapment neuropathies, trauma-induced neuropathies, poliomyelitis, and herpes zoster should be considered in the differential diagnosis [9]. The laboratory tests performed on our patient for vasculitis, an infective condition, autoimmune disease, and diabetes that may cause any neuropathy were normal. Pathologies seen in the cervical MRI were not evaluated as the main cause of the complaint because they were found to be incompatible with the patient's clinical findings in EMG correlation. Wrist and elbow MRIs, which we performed for a possible ulnar nerve entrapment neuropathy, were also reported as normal. MRI of the shoulder, which was performed to exclude rotator cuff tear, impingement, or bicipital tendinitis, was also reported as normal. In addition, a cranial MRI was requested to exclude a possible central nervous system event and was reported as normal. Lung radiographs and axillary ultrasonography ordered for any metastatic mass, lymphadenopathy, or fibrous band that may cause compression were also reported as normal. Doppler ultrasound was performed during Adson's maneuver to exclude thoracic outlet syndrome and was reported as normal. The control EMG performed indicated axonal damage and led us to the diagnosis of Parsonage-Turner as it was supported by clinical correlation.

Various presentations of Parsonage-Turner syndrome have been reported in the literature. Koca et al. have presented a case with bilateral axillary nerve involvement. They emphasized that a rare disease, Parsonage-Turner syndrome, should be considered in cases with sudden onset of severe pain and weakness in the shoulders and arms and highlighted the importance of EMG in diagnosis and treatment follow-up [3].

Fransz et al. reported a Parsonage-Turner case with shoulder pain and scapular winging while receiving prophylaxis against a possible HIV or HBV infection. They emphasized that the disease can be frequently confused with pathologies in the musculoskeletal system, especially in the neck and shoulder. They stated that the diagnosis is often delayed and takes an average of 10 weeks, and this syndrome should be considered in such patients because long thoracic nerve involvement is common in cases with scapular winging [7].

Terzi et al. also presented a case of Parsonage-Turner syndrome with recurrent shoulder pain and supraspinatus tendinitis. It was stated that the character of the pain should mainly be determined in patients with known rotator cuff lesions. Parsonage-Turner syndrome should be considered in patients with neuropathic pain, and the disease might also present with pre-existing shoulder pathology [2].

Weng and Fidel presented a case with dyspnea and isolated phrenic nerve involvement and reported that the disease might also present with visceral symptoms. Although rare, systemic steroid treatment given at the beginning of the treatment of the disease may be beneficial for pain improvement, does not affect recovery, and EMG is the most important diagnostic test [10].

## Conclusions

The clinical presentation of Parsonage-Turner syndrome, usually seen as a diagnosis of exclusion, settles in slowly. The disease should be carefully differentiated from cervical radiculopathy. The sudden onset of pain and its decrease over time, the observation of loss of muscle strength, and the fact that the pain does not change significantly with cervical movements and the Valsalva maneuver should prompt clinicians to consider a diagnosis of Parsonage-Turner syndrome. Parsonage-turner syndrome should be kept in mind in cases with sudden onset of back, shoulder, and upper extremity pain followed by weakness and sensory loss. Anamnesis and physical examination are the essential factors in diagnosing the disease, with EMG findings also playing a role in helping to make the diagnosis. Parsonage-Turner syndrome presenting with root involvement is very rare in the literature. Although root involvement is infrequent, it is definitely possible. Although rare, it should be kept in mind in cervical radiculopathies that do not improve despite treatment.

## References

[1] Parsonage MJ, Turner JW (1948). Neuralgic amyotrophy; the shoulder-girdle syndrome. Lancet.

[2] Terzi R, Yasar E, Yilmaz Z, Atlıhan D (2013). A rare cause of recurrent shoulder pain: parsonage turner syndrome (neuralgic amyotrophy). J PMR Sci.

[3] Koca I, Ibas E, Karagüllü H, Altindag O, Gür A (2011). The case of neuralgic amyotrophy with bilateral axillary nerve involvement. Turk J Phys Med Rehab.

[4] Feinberg JH, Radecki J (2010). Parsonage-Turner syndrome. HSS J.

[5] Ohta R, Shimabukuro A (2017). Parsonage-Turner syndrome in a patient with bilateral shoulder pain: a case report. J Rural Med.

[6] Fibuch EE, Mertz J, Geller B (1996). Postoperative onset of idiopathic brachial neuritis. Anesthesiology.

[7] Fransz DP, Schönhuth CP, Postma TJ, van Royen BJ (2014). Parsonage-Turner syndrome following post-exposure prophylaxis. BMC Musculoskelet Disord.

[8] Torres MO, Gudlavalleti A, Mesfin FB (2022). Brachial Plexitis. https://www.ncbi.nlm.nih.gov/books/NBK448114/.

[9] Akhtar N, Al Memar A (2009). Brachial neuritis presenting as isolated phrenic nerve palsy-two cases. J Neurol Neurosurg Psychiatry.

[10] Weng M, Fidel C (2010). Isolated unilateral brachial neuritis of the phrenic nerve (parsonage-turner syndrome) in a marathon runner with exertional dyspnea. Sports Health.

